# Home for Epileptic Boy

**Published:** 1911-09-23

**Authors:** 


### INSTITUTIONAL LETTER-BOX.

### HOME FOR EPILEPTIC BOY.

Our correspondent "H. H.," whose letter asking for information on the above subject was published in The Hospital of September 2 (page 586), in addition to the facts already brought to his notice, will be interested to learn that a "List of Institutions for the relief, cure, and care of persons suffering from paralysis, epilepsy, and other nervous diseases, and for incurable, imbecile, and feeble-minded persons," is published by the National Hospital for the Paralysed and Epileptic, Queen Square, London, W.C., and can be obtained from Mr. Godfrey H. Hamilton, the secretary, who is always willing to answer questions on the subject. The list, which is printed on one sheet of paper, gives all information as to conditions of admission and to fees, and should be studied side by side with "Burdetts' Hospitals and Charities," a copy of which can be obtained from this office.

